# Regulation Progression on Ellagic Acid Improving Poultry Production Performance by Regulating Redox Homeostasis, Inflammatory Response, and Cell Apoptosis

**DOI:** 10.3390/ani14203009

**Published:** 2024-10-17

**Authors:** Shengchen Wang, Wenjun Zhang, Bing Tian, Yun Hu, Tingting Li, Xiaoyan Cui, Liyang Zhang, Xugang Luo

**Affiliations:** 1 College of Animal Science and Technology, Yangzhou University, Yangzhou 225000, China; shengchenwang@yzu.edu.cn (S.W.); 19822629819@163.com (W.Z.);; 2 State Key Laboratory of Animal Nutrition, Mineral Nutrition Research Division, Institute of Animal Science, Chinese Academy of Agricultural Sciences, Beijing 100193, China

**Keywords:** medicinal homologous, poultry breeding, oxidative stress, inflammation, apoptosis

## Abstract

It has been approximately 2000 years since the medicinal homologous theory, which primarily holds that food has the same therapeutic value as medicine in order to improve the health of both humans and animals. In recent years, this theory has also been proposed to be used in poultry breeding. Ellagic acid (EA), a natural compound primarily extracted from medicinal homologous foods such as raspberries and pomegranates, is reported to have incomparable advantages in improving the production performance and disease resistance of poultry due to its pharmacological properties, which regulate the processes of redox homeostasis, inflammatory response, and cell apoptotic death. However, the application and research of EA in poultry production are still in the initial stage, and the potential mechanisms of its biological functions affecting animal health have not been clearly identified, which requires more attention worldwide. This mini-review collects the latest 10-year achievements of research on the effects of EA on poultry health, aiming to promote the practical application of EA in maintaining animal health and formulating corresponding targeted strategies.

## Simple Summary

Ellagic acid, extracted from medicinal foods like raspberries and pomegranates, enhances poultry production and disease resistance by regulating redox, inflammation, and cell death. However, the research on ellagic acid in poultry is nascent, with unclear mechanisms. This mini-review summarizes the latest 10-year findings to promote the practical use of ellagic acid in maintaining poultry health and formulating strategies.

## 1. Introduction

For the growth and health of livestock and poultry, oxidative stress, inflammation, and apoptosis occurring within tissue cells are important biological processes that have a significant impact on animal production performance and disease resistance, exhibiting complex interactions that depend on specific production and health environments [1,2]. Oxidative stress, typically characterized by the excessive production of free radicals, such as reactive oxygen species (ROS) and reactive nitrogen species (RNS), serves as a double-edged sword, initiating various diseases if unchecked [3]. Acute and chronic inflammation, two distinct responses to external stimulation like physical and chemical damages, pathogenic microorganisms, and autoimmune reactions, play crucial roles in both host defense and pathological progression [4]. Notably, the occurrence of oxidative stress can activate the signaling pathways related to the inflammatory responses and increase the level of proinflammatory cytokines and molecules, thereby triggering tissue inflammation [5,6]. Conversely, inflammatory stimulation exacerbates the occurrence of oxidative stress by inducing the generation of excessive free radicals, creating a vicious cycle between these two processes. Apoptosis, a highly regulated form of programmed cell death, relies on various signaling networks that converge on a comprehensive action mechanism [7]. The activation of apoptotic pathways is crucial for eliminating damaged cells within tissues and maintaining microenvironmental homeostasis [8]. Moreover, numerous studies have confirmed that the apoptotic signaling pathways can be activated or inhibited by oxidative stress and inflammation, leading to cell death in the end [6,9]. The intricate interdependence among oxidative stress, inflammation, and apoptosis is of paramount importance in livestock and poultry production. In cases where these processes are dysregulated, they contribute significantly to the pathogenesis of numerous disorders, impacting production performance, such as reduced growth rates, lower reproductive efficiency, and compromised meat quality, as well as decreasing disease resistance, thereby enhancing susceptibility to infections and inflammatory conditions [10,11,12]. Therefore, a comprehensive understanding of the molecular mechanisms underlying these biological processes, along with the identification of potential targets for intervention, is crucial for developing effective strategies to enhance livestock and poultry production performance and maintain physical health.

On account of the intimate link among redox homeostasis, the inflammatory response, and cell apoptotic death in biological systems, increasing research has been conducted on these properties of medicinal homologous foods, particularly their relevance to the production of livestock animals and poultry. These foods prominently embody the value of food and medicine fusion, and their unique biological activities that hold significant potential for enhancing animal health and productivity [13]. The bioactivities of medicinal homologous food extracts are closely related to their chemical compositions, including alkaloids, terpenoids, saponins, and flavonoids, which jointly promote their overall functional effects [14]. Ellagic acid (EA), a polyphenol substance found in natural resources like raspberries and pomegranates, mainly exists in its condensed form [15]. EA exhibits remarkable potential in the treatment of stress-induced pathologies in animals due to its ability to antagonize intracellular oxidative stress, inflammation, and cell apoptosis [16,17,18]. Meanwhile, the favorable safety profile and compatibility with other therapeutic agents of EA suggest its potential use as a natural additive or supplement in animal feeds, aiming to improve animal resilience and overall performance [19]. Consequently, the exploration of the potential application of EA in addressing oxidative stress-mediated inflammatory injury and cell apoptotic death in livestock and poultry represents a valuable theoretical framework and provides a promising avenue for enhancing animal welfare, health, and productivity. It is worth noting that there have been research results using poultry as model animals, which have found that EA plays an important positive role in improving poultry production performance and resisting harmful stimulation, providing a theoretical basis for its practical application in animal production.

## 2. The Chemical Properties of Ellagic Acid

Plant polyphenols are secondary metabolites derived from the metabolic pathways of shikimic acid and phenylalanine in plants, containing at least one phenolic hydroxyl group [20]. They can be divided into three categories: tannins, flavonoids, and lignin. EA belongs to the class of polyphenol extractives (tannins) widely spread among dicotyledons. Natural EA mainly exists in concentrated form in the ester linked to sugar in the hydrolyzable tannin component known as ellagitannins (ETs) [15]. During plant chemical processing, under either acidic or basic conditions, ester bonds of ETs are hydrolyzed, yielding a hexahydroxydiphenoyl group, which spontaneously lactonizes into EA [21]. The chemical formula of EA is C_14_H_6_O_8_, with a molecular weight of 302.19 g/mol, known as 2,3,7,8-tetrahydroxy-chromeno [5,4,3-cde]chromene-5,10-dione. Pure EA is a milky white crystal with a melting point above 360 °C. It is a highly symmetrical molecule, where one molecule of EA is composed of hydrophilic parts of four hydroxyls and two lactones functional groups and lipophilic parts of four six-membered hydrocarbon rings, resulting in the whole molecular space maintaining a planar distribution [22]. Due to the interaction of hydroxyl groups and the lactone systems forming hydrogen bonds, functions such as participating in antioxidant redox reactions are performed by EA [23]. Under the influence of its structure, EA shows poor aqueous solubility, which is the major pharmacological limitation; however, it is slightly soluble in methanol, alcohol, and dimethyl sulfoxide, and the best solvent is polyethylene glycol [24,25].

## 3. The Anti-Oxidant Property of Ellagic Acid

Oxygen free radicals, especially ROS, are generated intracellularly within the mitochondria and other cellular components [26,27]. Under physiological conditions, low levels of ROS are crucial for maintaining many important physiological functions by regulating redox homeostasis and key signaling pathways. When the ROS accumulates excessively, they can cause the occurrence of oxidative stress and destroy all of the key biomolecules, including proteins, membrane lipids, and DNA, ultimately leading to various diseases such as intestinal inflammation and digestive dysfunction, immunologic suppression, reproductive disorders, and muscle dysplasia and growth retardation in livestock animals and poultry [28,29,30,31]. The characteristic of oxidative stress is the transition from the balance between the formation and elimination of free radicals to their excessive formation. To alleviate the adverse effect of ROS, organisms have evolved a sophisticated and multilevel antioxidant defense system, in which the initial and most effective layer comprises enzymes with superior catalytic activity, including glutathione peroxidases (GPx), superoxide dismutases (SODs), thioredoxin, and catalase (CAT), and some antioxidant substrates [32,33]. This intricate system efficiently protects cells from ROS-induced toxicity and alleviates the impairment caused by oxidative stress [34].

Known for its remarkable anti-oxidative stress characteristics, EA stands out as a naturally obtainable pharmacological agent, with a bioactive polyphenolic molecule structure. Numerous studies from both in vivo and in vitro trials have consistently demonstrated the protective function of EA in alleviating oxidative damage in animals caused by various adverse factors, such as heat stress, dietary changes, and hazardous substance exposure [35]. The antioxidant function of EA is achieved by either attenuating the accumulation of excessive ROS or activating specific antioxidant enzymes [36,37]. A previous study showed that dietary supplementation with EA (100, 200, or d 400 mg/kg) could improve serum GPx activity, increase intestine villus height and crypt depth, and decrease the feed/gain ratio in yellow-feathered broilers [38]. Meanwhile, the study result on the potential effects of EA and mesocarp extract of *Punica granatum* on the productive and reproduction performance of laying hens indicated that the supplementation of EA or *Punica granatum* extract to diet remarkably restrained malondialdehyde (MDA) concentration and increased total antioxidant capacity in serum and liver samples, accompanied with increased egg production, feed intake, and body weight of hens [39]. Furthermore, the addition of different doses of EA (0, 75, 150, 300, and 600 mg/kg) to the basic diet can significantly increase the activities of antioxidant enzymes GPx, SOD, and CAT and reduce the level of peroxide MDA in the serum of Arbor Acres broilers under heat stress, thus improving intestinal barrier function and growth performance parameters (Final weight and the feed/gain ratio) [35].

**Figure 1:**
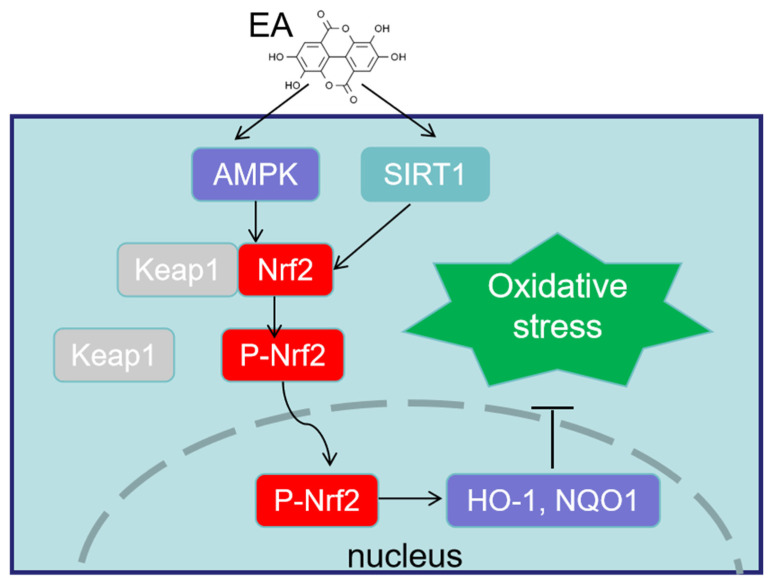
The mechanism of ellagic acid EA in improving the antioxidant system function of poultry. EA = ellagic acid; SRIT = sirtuin 1; AMPK = AMP-activated protein kinase; Keap1 = kelch-like ECH-associated protein 1; Nrf2 = nuclear factor erythroid 2-related factor 2; P-Nrf2 = phosphorylated Nrf2; HO-1 = heme oxygenase-1; NQO1 = quinone oxidoreductase 1.

Interestingly, the molecular mechanism by which EA activates the antioxidant system has also been extensively studied and confirmed. It is reported that nuclear factor erythroid 2-related factor 2 (Nrf2) is the key target for EA to stabilize ROS levels [40,41]. As a classical nuclear receptor transcription factor, Nrf2 is a crucial regulator of redox homeostasis. Under quiescent conditions, two glycine repeat domains of kelch-like ECH-associated protein 1 (Keap1) homodimer bind with the latch and the hinge domains of a single Nrf2 molecule to repress Nrf2 activity; however, when cells are exposed to stress resources, the glycine repeat domains of Keap1 released the hinge domain of Nrf2 to block Nrf2 ubiquitination and degradation, thus inducing free and newly synthesized Nrf2 to translocate to the nucleus to achieve its active function [42,43]. Recent studies have demonstrated that EA can directly stimulate the nuclear translocation of Nrf2, enhancing its interaction with the antioxidant response element sequence found in antioxidant enzymes such as heme oxygenase-1 (HO-1) and quinone oxidoreductase 1 (NQO1) [31,44]. This process increases the expression of these enzymes, which, in turn, helps to alleviate oxidative stress and offers a defensive and protective mechanism against the detrimental effects of xenobiotic metabolism [45,46]. For instance, it has been reported that supplementing with EA in orally administered *Lactobacillus brevis* can significantly increase the mRNA and protein expression of Nrf2, promote the mRNA expression levels of HO-1, NQO1, and GPx1, and increase the contents of SOD, CAT, and glutathione in the caeca of Leghorn chickens [47]. Meanwhile, a recent study has shown that EA has the ability to exert protective effects on tissue dysfunction and structural alteration through the improvement in antioxidant function regulated by sirtuin 1 (SIRT1) [48]. SIRT1 is a histone deacetylase that can sense energy levels in cells and has the function of delaying cell aging, helping cells resist external stress, and improving metabolism. It has been reported that SIRT1 activates Nrf2 nuclear translocation through deacetylation, thereby regulating the expression of its downstream antioxidant and detoxification gene [49]. In addition, besides SIRT1, AMP-activated protein kinase (AMPK) is also targeted and inhibited by EA to alleviate LPS-induced oxidative stress [50]. AMPK can phosphorylate Nrf2 or its upstream signaling molecules, indirectly enhancing the defense ability of the antioxidant system [51]. Therefore, based on existing evidence, activating Nrf2 activity is a key mechanism for EA to exert antioxidant function and maintain tissue cell redox homeostasis (Figure 1). In conclusion, an in-depth understanding and exploration of the regulatory mechanism of EA on redox homeostasis provide substantial help for the subsequent use of EA in preventing adverse consequences related to oxidative stress.

## 4. The Anti-Inflammatory Property of Ellagic Acid

Inflammation, a highly conserved and sophisticated defense mechanism, serves as a crucial response to diverse stimulations ranging from microbial invasion, mechanical trauma, or chemical insults, and its primary objective is to neutralize the source of damage and reinstate tissue homeostasis, which is important for maintaining animal health and performance [52]. As a response to these stimuli, immune cells in injured tissues release proinflammatory cytokines, chemokines, and inflammatory metabolites, collectively known as inflammatory mediators [53]. These mediators play an important role in orchestrating the inflammatory response, which is indispensable for combating infections and facilitating tissue repair. However, once the delicate balance between the generation and elimination of inflammatory mediators is disrupted, the tissue will experience sustained or excessive inflammation in livestock and poultry. This dysregulation can develop into different types of diseases, such as intestinal lesions that impair nutrient absorption, metabolic disturbances affecting growth and reproduction, and autoimmune disorders that compromise overall health and welfare [54,55]. Modulating inflammatory mediators emerges as a strategic approach for preventive and therapeutic interventions in livestock and poultry. By targeting specific inflammatory mediators or pathways, researchers aim to mitigate the detrimental consequences of excessive inflammation, enhance animal resilience against disease challenges, and ultimately optimize production efficiency and animal health outcomes.

As a substance endowed with the ability to regulate numerous chemicals and signaling pathways that are involved in the inflammatory cascade, EA exhibits a remarkable potential to modulate the inflammatory response. A series of studies have shown that the reason why EA exerts anti-inflammatory effects is attributed to inhibiting the actions of several mediators that promote inflammation, such as proinflammatory cytokines (interferon γ [IFN-γ], interleukin [IL]-1, 1 IL-6, and IL-17) and chemokines (C-C motif ligand [CCL]-2 and CCL-3), or promote the release of the anti-inflammatory cytokine-like IL-10 [56,57,58]. For example, dietary EA significantly reduced the content levels of IL-6 and TNF-α and increased the levels of IL-2, IL-10, and immunoglobulins IgG, IgM, and IgA in the cecal tissue of Guangxi yellow feathered broiler chickens infected with *Eimeria tenella* [59]. Of note, the nuclear factor kappa-light-chain-enhancer of activated B cells (NF-κB) pathway, being crucial in the inflammatory process, has been shown to be effectively blocked by EA [60]. NF-κB coordinates the expression of crucial regulatory genes through two distinct activation mechanisms, namely the canonical and noncanonical pathways [61]. The canonical pathway of NF-κB, triggered by inflammatory and immune signals, utilizes the IκB kinase (IKK) complex (IKKγ, IKKα, and IKKβ) to phosphorylate IκB, releasing NF-κB dimers to regulate transcription of proinflammatory cytokines such as tumor necrosis factor-α (TNF-α) and IL-1 [62,63]. EA mainly alleviates inflammation via the NF-κB canonical pathway. Previous studies have demonstrated that by targeting the IKK-NF-κB pathway, EA could lead to a reduction in the expression of these proinflammatory cytokines and retards the subsequent inflammation occurrence [64,65]. It was reported that under post-infection with *Eimeria tenella*, significant anti-inflammatory effects were observed in chickens orally gavaged with EA and in combination with *Lactobacillus*, which was accompanied by NF-κB pathway expression inhibition [47].

**Figure 2:**
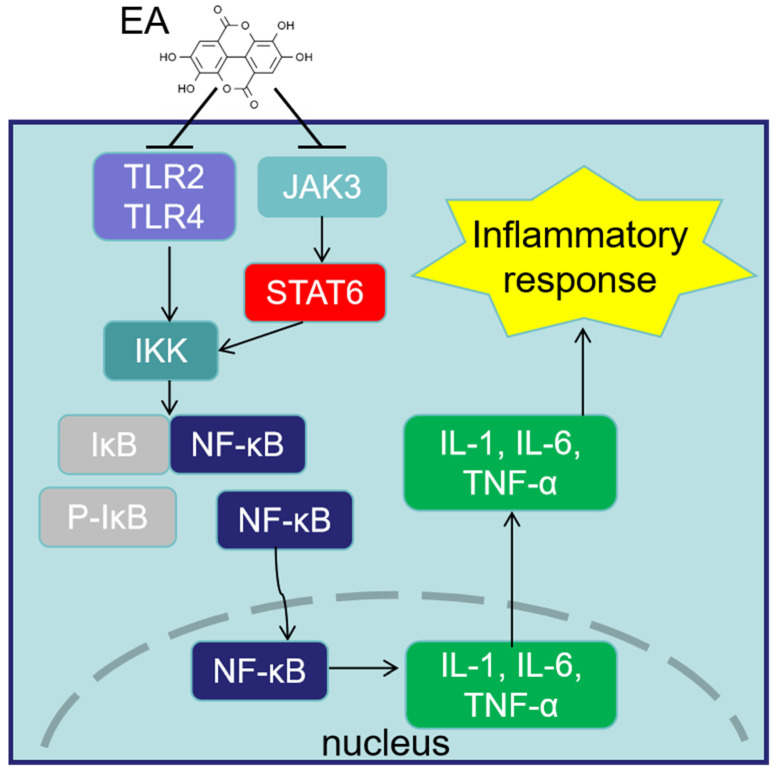
The mechanism of ellagic acid in suppressing the inflammatory response of poultry. EA = ellagic acid; JAK3 = Janus kinase 3; STAT6 = signal transducers and transcriptional activator 6; TLR2 = toll-like receptor 2; TLR4 = toll-like receptor 4; NF-κB = nuclear factor kappa-light-chain-enhancer of activated B cells; IκB = inhibitor of NF-κB; IKK = IκB kinase; P-IκB = phosphorylated IκB; IL-1 = interleukin 1; IL-6 = interleukin 6; TNF-α = tumor necrosis factor-α.

Furthermore, some upstream signaling molecules or pathways have also been reported to be regulated by EA, thereby affecting NF-κB pathway-mediated inflammation processes. For instance, in mouse tissue injury models (such as ulcerative colitis, hepatitis, and neuralgia), EA administration can alleviate NF-κB-activated tissue inflammation and pathological damage by inhibiting the phosphorylation activation of the mitogen-activated protein kinase pathway (c-Jun N-terminal kinase, extracellular regulated kinase 1/2, and p38) or the expression of toll-like receptors (TLR2 and TLR4) [66,67]. Significantly, the NF-κB dimer activates target genes by binding to specific DNA binding sites, often requiring the assistance of other transcription factors. For instance, the signal transducers and transcriptional activators (STAT) family can directly control the transcription expression of NF-κB to participate in the inflammation occurrence [68]. The six primary isoforms of the STAT family (STAT1 to STAT6) play significant roles in regulating signal transduction involved in cell proliferation and death, immunity reaction, and metabolism [69]. The ability of EA to inhibit the expression of the STAT transcription factor family is believed to regulate the inflammatory response [70]. Studies have shown that EA can inhibit the activation of Janus kinase (JAK), thereby limiting the recruitment and phosphorylation of STAT protein and alleviating NF-κB-mediated inflammation [62,71]. Tang et al. investigated the therapeutic effects of dietary EA on subclinical necrotic enteritis in broilers and found that EA could alleviate intestinal barrier dysfunction and inflammatory damage in the jejunum, as well as decrease the feed conversion ratios of broilers by modulating the TLR/NF-κB and JAK3/STAT6 signaling pathways [72]. These studies clearly explain the mechanism of EA influencing the NF-κB pathway and its cascade of events (Figure 2), which contributes to emphasizing its potential use as a medication to treat a range of inflammatory conditions in poultry production.

## 5. The Anti-Apoptosis Property of Ellagic Acid

Apoptosis, universally recognized as a type of programmed cell death, constitutes a crucial biological mechanism essential for maintaining tissue homeostasis by meticulously regulating cell populations. This process selectively eliminates dysfunctional, damaged, or superfluous cells, thereby ensuring the overall health and stability of an organism [73,74]. The occurrence of apoptosis is governed by two primary signaling pathways: the extrinsic and intrinsic pathways [75]. The extrinsic pathway, also known as the death receptor pathway, is initiated when specific ligands bind to transmembrane death receptors (such as Fas) on the cell surface, triggering a cascade of signaling events that ultimately lead to apoptosis [76]. In contrast, the intrinsic pathway is activated in response to intracellular stress signals, such as oxidative stress and inflammation, ultimately leading to permeabilization of the outer mitochondrial membrane and releasing proapoptotic factors that further propagate the apoptotic signaling [7]. The final execution of apoptosis relies on the caspase family of proteases, which are activated by both the extrinsic and intrinsic pathways. These proteolytic enzymes function as molecular scissors, cleaving key cellular substrates to dismantle the cells in a highly energy-dependent manner [77]. However, under the influence of excessive oxidative stress and uncontrolled inflammation, damaged tissues can also undergo excessive apoptosis, which can have a negative impact on the normal physiological functions of livestock and poultry, thereby endangering their production performance [78].

EA can maintain cell viability by inhibiting the activation of pro-apoptotic pathways. In a rat liver injury model induced by methotrexate exposure, oral administration of EA significantly activated the expression of antioxidant genes Nrf2 and HO-1 and inhibited the expression of proinflammatory factors NF-κB and IL-6, thereby alleviating methotrexate-induced mitochondrial dysfunction and apoptosis in liver cells [79]. Moreover, EA treatment significantly downregulated the STAT3/NF-κB pathway and improved testosterone propionate-induced benign prostate hyperplasia in mice by decreasing the inflammatory response, increasing mitochondrial dynamics, and suppressing apoptotic death [62]. Therefore, it can be concluded that EA mainly reduces tissue cell apoptosis caused by pathogenic stimuli in various diseases by exerting its biological effects of antioxidant and anti-inflammatory effects. In the field of poultry production, research on the anti-apoptotic potential of EA is still at a nascent stage. Encouragingly, in a quail model of lead poisoning, the supplementation of EA in diets could significantly alleviate the adverse effects of lead on the growth performance parameters of laying quails (*Coturnix japonica*) and suppress apoptosis in the liver tissue by enhancing the antioxidant defense system [80]. Meanwhile, the results from in vitro cellular experiments have demonstrated that after storing the semen of ROSS 380 roosters in Beltsville extender containing EA liposomes for a certain period of time, compared with the control, EA liposomes significantly increased the GPx and total antioxidant capacity of sperm and reduced the rate of apoptosis, thus accompanying an increase in total motility and membrane functionality [81]. Therefore, we have reason to believe that dietary supplementation with EA has the ability to help poultry reduce the occurrence of abnormal apoptosis of tissue cells. However, whether the anti-apoptotic role of EA in poultry farming to maintain tissue and cellular health is universal remains to be confirmed through extensive practical applications.

## 6. Conclusions and Perspectives

**Figure 3:**
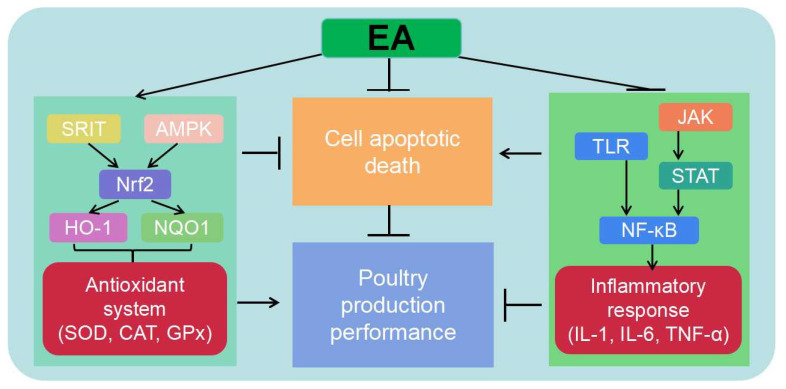
A graphic summary of ellagic acid in improving poultry production performance via regulation of redox homeostasis, the inflammatory response, and cell apoptosis. EA = ellagic acid; SRIT = sirtuin 1; AMPK = AMP-activated protein kinase; Nrf2 = nuclear factor erythroid 2-related factor 2; HO-1 = heme oxygenase-1; NQO1 = quinone oxidoreductase 1; SOD = superoxide dismutases; CAT = catalase; GPx = glutathione peroxidases; JAK = Janus kinase; STAT = signal transducers and transcriptional activators; TLR = toll-like receptor; NF-κB = nuclear factor kappa-light-chain-enhancer of activated B cells; IL-1 = interleukin 1; IL-6 = interleukin 6; TNF-α = tumor necrosis factor-α.

EA, a bioactive compound present in various plants, performs significant roles in the prevention of diseases, such as immune-related diseases, intestinal injury, and metabolic disorders. This mini-review summarizes an overview of its recent research progress in preventing and treating oxidative stress, inflammation, and cell apoptosis in poultry production, and provides relevant reports on how it helps poultry improve production performance and resist harmful stimuli by regulating these biological processes. Generally, EA mainly exhibits antioxidant properties through Keap1/Nrf2 pathway activation, anti-inflammation properties through NF-κB pathway inhibition, and reduces the incidence of abnormal apoptotic death under the dynamic changes in these two key biological processes (Figure 3). EA has broad application prospects in animal production, including the development of new drugs in the context of antibiotic-free feeding, exploration of alternative treatment methods, clinical trials of targeted therapies for specific diseases, and the combination of feeding interventions to maintain overall health. However, advancing research requires a comprehensive understanding of the mechanisms of EA in reducing diseases and the development of more effective nutritional regulation strategies.
